# Initial decrease in the ambient dose equivalent rate after the Fukushima accident and its difference from Chernobyl

**DOI:** 10.1038/s41598-020-60847-0

**Published:** 2020-03-02

**Authors:** Kazuya Yoshimura, Jun Saegusa, Yukihisa Sanada

**Affiliations:** 1 Fukushima Environmental Safety Center, Japan Atomic Energy Agency, 45-169 Sukakeba, Minamisoma, Fukushima 975-0036 Japan; 2 Collaborative Laboratories for Advanced Decommissioning Science, Japan Atomic Energy Agency, 4-33, Muramatsu, Tokai−mura, Ibaraki, 319-1194 Japan

**Keywords:** Environmental sciences, Health care

## Abstract

In 2011, after the Fukushima Dai-ichi Nuclear Power Plant accident, the initial decrease in the ambient dose equivalent rate (dH*(10) *dt*^−1^), an alternative quantity to the effective dose, was studied using monitoring data obtained from March 16, 2011. The dH*(10) *dt*^−1^ was normalized by the ^137^Cs activity per unit area (norm-dH*(10) *dt*^−1^) to analyze the data across monitoring sites with different deposition levels. The norm-dH*(10) *dt*^−1^ showed a rapid decrease during the first 60 days, followed by slow decrease and was modeled using two exponential functions. The norm-dH*(10) *dt*^−1^ obtained in areas dominated by paved surfaces and buildings showed a faster decrease than the unpaved-dominant field, and this decrease was facilitated in residential areas compared with the evacuation zone. The decrease in norm-dH*(10) *dt*^−1^ was compared with simulation results using parameters obtained in Europe after the Chernobyl Nuclear Power Plant accident that represent a decrease due to radionuclide migration (e.g., soil penetration and horizontal wash-off). The simulation results showed a faster decrease than our results, implying that there was less radiocesium migration in Fukushima than in Europe. The results also suggested that the regional variation in the decrease rate led to uncertainty regarding the external dose estimation.

## Introduction

The radiation exposure level is essential information for measures of radiation protection such as evacuation orders, decontamination planning, and retrospective risk analyses following a major radionuclide-release incident. Intergovernmental organizations, such as the United Nations Scientific Committee on the Effects of Atomic Radiation (UNSCEAR) and the World Health Organization, have simulated the effective dose in detail for the areas affected by the Fukushima Dai-ichi Nuclear Power Plant (FDNPP) accident in consideration of typical exposure pathways^1,2^. The simulation demonstrates that groundshine was the major pathway and that the first month had a larger influence on the total dosage than the subsequent three months together, meaning that the external dose during the initial period after such an accident is critical for radiation protection.

To calculate the external dose, the simulation considers the behavior patterns of residents (occupancy factor) as well as the reduction of gamma rays in buildings (shielding factor). Furthermore, the simulation reflects temporal decreases in gamma rays in fields due to the decay of radionuclides, soil penetration of ^137^Cs (termed the attenuation function (*AF*))^3–5^, and weathering effects (i.e., horizontal migration due to wash-off) of ^137^Cs depending on the location (termed the location factor (*LF*))^4–6^. The shielding^7–10^ and occupancy factors^8,11–13^, their variations and influences on the external dose estimate have been well documented since the FDNPP accident. However, the *AF* and *LF* have been based on knowledge primarily obtained in Europe after the Chernobyl Nuclear Power Plant (CNPP) accident, and the factors related to gamma ray decreases due to the migration of radionuclides have rarely been investigated in the other case. As variations in the *AF* and *LF* directly relate to the uncertainty of the external dose estimation, it is necessary to evaluate these variations for radiation protection.

The distribution of the dH*(10) *dt*^−1^, an alternative quantity to the effective dose, over an extensive area has been monitored from June 2011 by a national project since the FDNPP accident^14^. This comprehensive monitoring provides important information regarding aspects, such as the effects of land use and human activities, on the decrease in the dH*(10) *dt*^−1^ ^15–18^. However, the monitoring measurements are only performed once or twice each year. Therefore, information on the initial decrease in the dH*(10) *dt*^−1^ during the initial period after the accident and the temporal resolution of the data are limited, even though the immediate period after the accident had a large influence on the external exposure. We monitored the dH*(10) *dt*^−1^ at a maximum of 103 points located outside a 20-km radius from the FDNPP since March 15, 2011, as part of a monitoring program of the Ministry of Education, Culture, Sports, Science and Technology (MEXT) and Nuclear Regulation Authority^19,20^. In this monitoring program, the dH*(10) *dt*^−1^ and other additional information, such as weather conditions, decontamination activities, and ground surface changes, were recorded either every day or every week in 2011. These datasets with fine temporal resolution have enabled the characterization and evaluation of the contributing factors of the decrease in the dH*(10) *dt*^−1^.

This study aims to characterize the decrease in the dH*(10) *dt*^−1^ during the initial period after the accident using the *in-situ* monitoring data obtained by the program in 2011. Additionally, the monitoring data are compared with simulation results based on the data obtained in Europe after the CNPP accident to determine the factors affecting the decrease in the dH*(10) *dt*^−1^ and evaluate the impact of variations in the *AF* and *LF* on the external dose estimation.

## Results and Discussion

### Dataset arrangement

**Figure 1:**
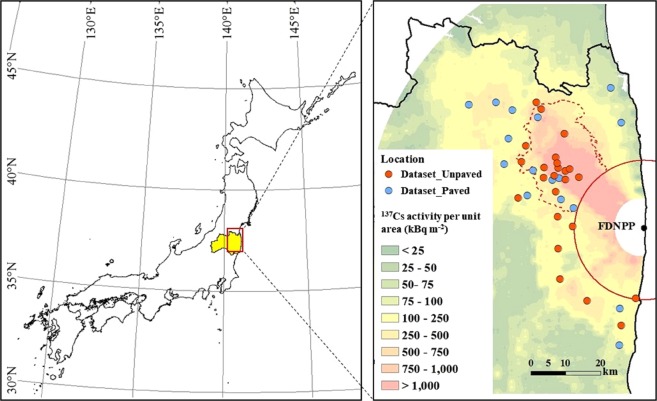
Location of the monitoring points with ^137^Cs activity per unit area as of the value on July 01, 2011, obtained by MEXT^41^. Both the red dashed and solid lines indicate the borders of the evacuation zones (termed the deliberate evacuation areas and restricted areas, respectively) at the end of 2011. This map was created using ArcMap 10.6 (https://desktop.arcgis.com/en/arcmap/).

Of the 103 monitored points, the data obtained at decontaminated sites (54 points) and sites showing low and constant dH*(10) *dt*^−1^ values of less than 0.1 μSv h^−1^ due to little radionuclide deposition (nine points) were excluded from the analysis. The data collected at the remaining 40 points were analyzed after excluding the data collected during rainy conditions and periods of snow cover. Then, the data were categorized into two datasets as follows: Dataset_Unpaved (≥50% covered by grassland and bush, *N* = 24) and Dataset_Paved (>50% covered by paved surfaces and buildings, *N* = 16). The locations of the measurement points are shown in Fig. 1, and their descriptions are summarized in Supplementary Tables S1 and S2. Most of the points were located northwest of the FDNPP, an area that was largely affected by the wet deposition of radionuclides on March 15, 2011^21,22^. Half of the monitoring points were located in the evacuation zone throughout 2011.

### Characteristics of the temporal decrease in the norm-dH*(10) *dt*^−1^

**Figure 2:**
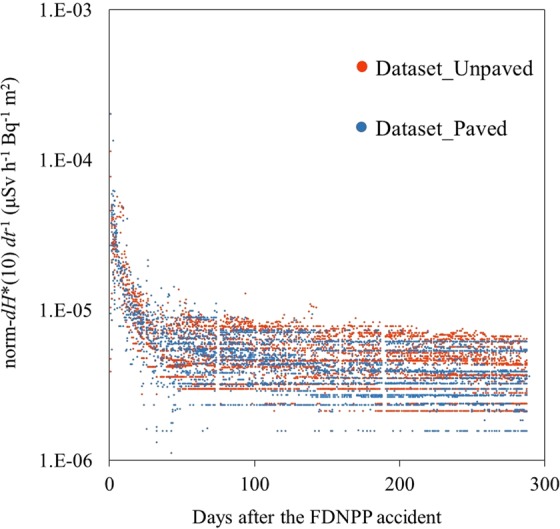
Trend in the norm-dH*(10) *dt*^−1^ though time observed for both the unpaved-surface dominant (Dataset_Unpaved) and paved-surface dominant (Dataset_Paved) areas.

To analyze the data across monitoring sites with different deposition levels, the dH*(10) *dt*^−1^ was normalized by dividing it via the deposition amount of ^137^Cs (Bq m^−2^) on March 15, 2011 (norm-dH*(10) *dt*^−1^). The temporal decreases in the norm-dH*(10) *dt*^−1^ are shown in Fig. 2. The norm-dH*(10) *dt*^−1^ showed similar temporal trends between the two datasets (i.e., rapid decreases during the first 60 days) followed by slower decreases.

The temporal decrease in the norm-dH*(10) *dt*^−1^ with two phases of decrease was represented as follows:

1 $$norm-d{H}^{\ast }(10)\,d{t}^{-1}(t)={a}_{0}\times exp(-{k}_{1}\times t)+{b}_{0}\times exp(-{k}_{2}\times t)$$

where *a*_0_ and *b*_0_ are constants representing the initial fractions of the norm-dH*(10) *dt*^−1^ (μSv h^−1^ Bq^−1^ m^2^), which decreased rapidly and slowly over time *t* (year) with decrease rates of −*k*_1_ and −*k*_2_ (year^−1^), respectively. These rates included the radionuclide decay. The obtained parameters are summarized in Table 1. Dataset_Paved showed faster decrease rates (−*k*_1_ and −*k*_2_) than Dataset_Unpaved. A larger proportion of the rapidly-decreasing fraction (*a*_0_/*b*_0_) was also found for Dataset_Paved. The long-term monitoring^15–18^ showed that the decrease in the dH*(10) *dt*^−1^ was faster in residential areas than other land uses. The results also demonstrated that the decrease in the norm-dH*(10) *dt*^−1^ was facilitated in the paved-dominant field, even in the initial year following the accident. The radiocesium on paved surfaces has been suggested to have been removed by weathering effects faster than that on unpaved ground after both the CNPP^23,24^ and FDNPP accidents^25^. The difference in the radionuclide wash-off could be one reason explaining the faster decrease rate of the norm-dH*(10) *dt*^−1^ in paved-dominant fields.

**Table 1:** Parameters in Eq. (1) obtained for both Dataset_Unpaved and _Paved.

	*N*	*a*_0_	−*k*_1_	*b*_0_	−*k*_2_
**Dataset_Unpaved**					
All data	24	4.4 × 10^−5^ (4.2 ~ 4.6 × 10^−5^)	46 (49 ~ 43)	6.0 × 10^−6^ (5.6 ~ 6.3 × 10^−6^)	0.43 (0.56 ~ 0.30)
Evacuation zone	15	4.3 × 10^−5^ (4.1 ~ 4.5 × 10^−5^)	46 (49 ~ 43)	6.4 × 10^−6^ (6.0 ~ 6.7 × 10^−6^)	0.36 (0.49 ~ 0.23)
Residential area	9	4.5 × 10^−5^ (4.2 ~ 5.0 × 10^−5^)	46 (53 ~ 40)	5.4 × 10^−6^ (4.8 ~ 6.1 × 10^−6^)	0.45 (0.70 ~ 0.20)
**Dataset_Paved**					
All data	16	5.7 × 10^−5^ (5.3 ~ 6.1 × 10^−5^)	65 (72 ~ 60)	6.6 × 10^−6^ (6.1 ~ 7.0 × 10^−6^)	0.91 (1.08 ~ 0.74)
Evacuation zone	5	2.4 × 10^−5^ (2.1 ~ 2.6 × 10^−5^)	36 (40 ~ 32)	5.7 × 10^−6^ (5.3 ~ 6.0 × 10^−6^)	0.54 (0.67 ~ 0.40)
Residential area	11	6.2 × 10^−5^ (5.7 ~ 6.9 × 10^−5^)	72 (83 ~ 63)	6.9 × 10^−6^ (6.3 ~ 7.6 × 10^−6^)	1.05 (1.28 ~ 0.82)

The parameters were also obtained for the dataset divided into evacuation zone and residential area, respectively. The *N* indicates number of data, and ranges in the parenthesis represent 95% confidential intervals.

Human activities have also been reported to be an important factor facilitating the decrease in the dH*(10) *dt*^−1^ based on long-term monitoring results^15–18^. To evaluate the effect of human activities on the initial decrease in the norm-dH*(10) *dt*^−1^, Eq. (1) was applied to the datasets in both the evacuation zone and residential areas. The obtained parameters are summarized in Table 1. Although no clear differences in the decrease rates between the evacuation zone and residential areas were found for Dataset_Unpaved, the residential areas of Dataset_Paved showed larger −*k*_1_ and −*k*_2_ to the evacuation zone. Dataset_Unpaved was collected in an unpaved-dominant field without buildings and other artificial structures, suggesting rural or forested fields and limited human activities. Meanwhile, Dataset_Paved was collected in an urbanized area wherein human activities were expected outside the evacuation zone. Therefore, the differences in the −*k*_1_ and −*k*_2_ between the evacuation zone and residential areas of Dataset_Paved were provably controlled by human activity, consistent with other long-term monitoring^15–18^. Both the *−k*_1_ and −*k*_2_ in the residential area were double those in the evacuation zone, suggesting that human activities could largely reduce external radiation exposure, especially in urbanized areas. However, human activities such as traffic and agricultural practices could increase radioactivity in the air due to the resuspension of dust^26,27^, associating to which is associated with an increase in inhalation doses. To better understand the effect of human activity on the entire effective dose, further comprehensive investigations are necessary.

### Simulation of the norm-dH*(10) *dt*^−1^

In this study, the time dependency of the norm-dH*(10) *dt*^−1^ due to decay, *AF*, and *LF* was simulated according to UNSCEAR^1^, which applied parameters obtained in Europe after the CNPP accident. Following reports by UNSCEAR^1^, the simulation assumed radionuclide/^137^Cs ratios of ^131^I = 11.5, ^132^I (radioactive equilibrium with ^132^Te) = 8, ^134^Cs = 1, ^136^Cs = 0.17, ^110 m^Ag = 0.0028, and ^129 m^Te = 1.1. The radionuclide composition was regionally variable; in particular, larger proportions of ^131^I, ^132^I, ^132^Te, and ^129 m^Te than the above values were found in the area south of FDNPP^1^. However, most of the data analyzed in this study were located northwest of the FDNPP (Fig. 1), and the regional variations in the radionuclide composition scarcely affected the comparison between the simulation result and models of decrease in the norm-dH*(10) *dt*^−1^ in this study.

**Figure 3:**
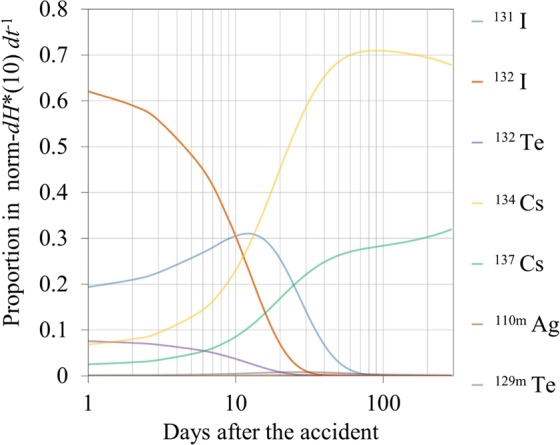
Proportion of each nuclide’s contribution in the simulated norm-dH*(10) *dt*^−1^ considering the decrease due to decay only.

To better understand the contribution of radionuclides to the norm-dH*(10) *dt*^−1^, the time dependence of the contributions was simulated by considering decay only (Fig. 3). A major proportion was derived from ^131^I and ^132^I just after the accident, but their contributions steeply decreased 60 days later. The norm-dH*(10) *dt*^−1^ was primarily derived from the ^134^Cs and ^137^Cs during the latter period as reported^1^.

### Differences in the decreases due to radionuclide migration

**Figure 4:**
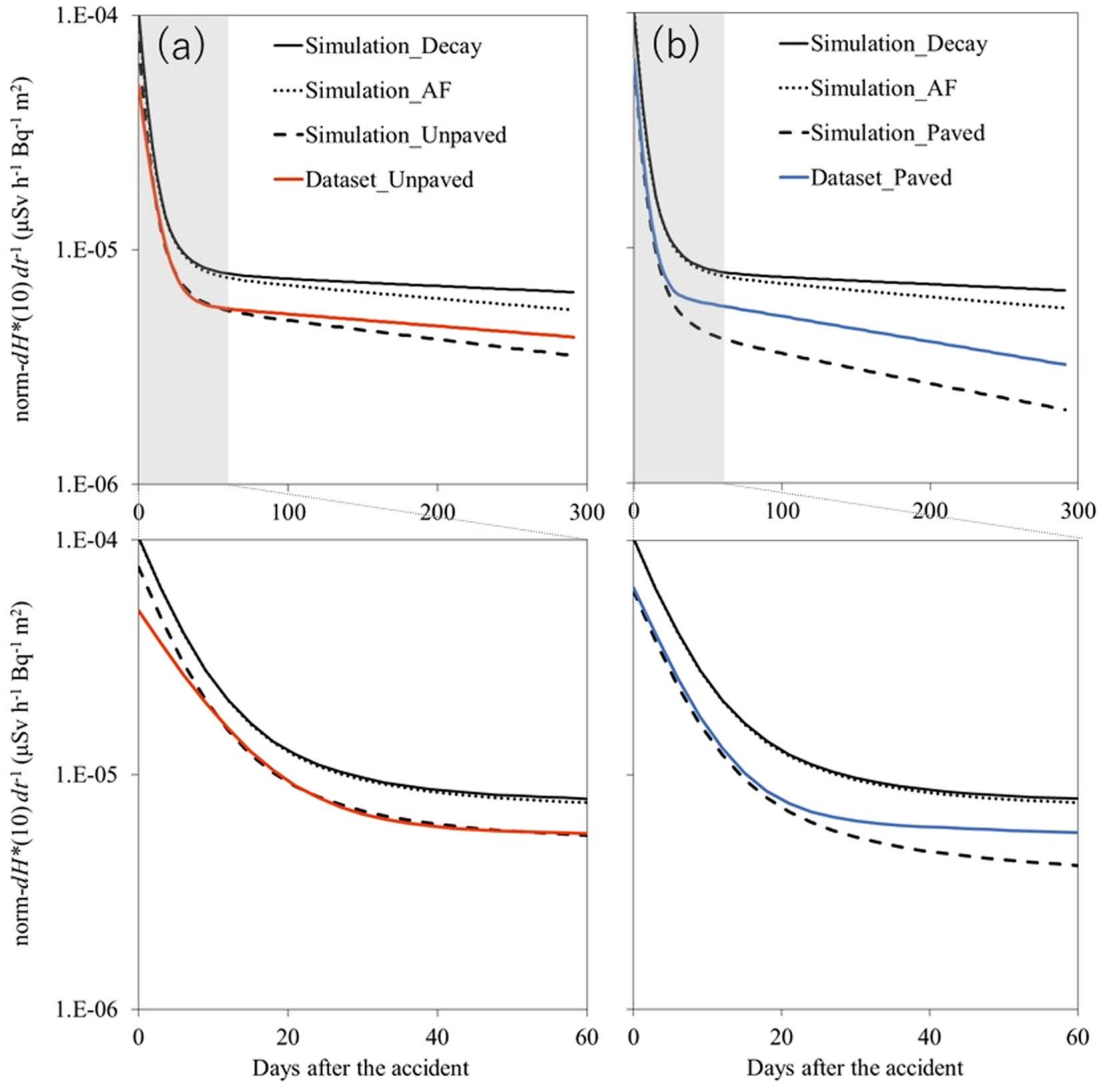
Temporal decrease in the norm-dH*(10) *dt*^−1^ simulated according to UNSCEAR^1^ using parameters obtained in Europe after the CNPP accident (black lines) and modeled in this study (colored lines). The gray area in the upper figure indicates the initial 60-day period. The simulation and model were applied to both the (**a**) unpaved- and (**b**) paved-dominant fields. This study simulated three cases of decrease in the norm-dH*(10) *dt*^−1^: Case 1: decrease due to decay only (Simulation_Decay (solid line)); Case 2: decrease due to decay and radionuclide penetration into the ground (i.e., *AF*) (Simulation_*AF* (dotted line)); and Case 3: decrease due to decay, *AF*, and radionuclide washout (i.e., *LF*) (Simulation_Unpaved and Simulation_Paved (dashed line)).

The simulation results of the decrease in the norm-dH*(10) *dt*^−1^ are shown in Fig. 4 with the Dataset_Unpaved and Dataset_Paved models obtained in this study. Although the result of the Simulation_Unpaved slightly overestimated the norm-dH*(10) *dt*^−1^ during the initial period, the simulated norm-dH*(10) *dt*^−1^ for both _Unpaved and _Paved showed rapid decreases similar to those in Dataset_Unpaved and Dataset_Paved during the first 60 days, respectively. These rapid decreases were mainly caused by the radionuclide decay, as represented by Simulation_Decay; short half-life radionuclides such as ^131^I and ^132^I were the major radionuclides of the rapid decrease, as shown in Fig. 3. However, the models showed a different trend from the simulation results after 60 days, displaying slower decreases than those in the simulation. In particular, the decrease rate of Dataset_Unpaved was smaller than that of Simulation_Unpaved, even though the monitoring sites of the former included a certain proportion of paved surfaces that may have facilitated the decrease. The slower decreases in the models in this study indicated that the radionuclide migration was smaller in Fukushima than in Europe after the CNPP accident.

To better understand the factors contributing to the decrease in the norm-dH*(10) *dt*^−1^ in Fukushima during the later phase, an exponential function was fitted to the models and simulations after June 2011 (78 days after the accident) in Fig. 4. The decrease rates are summarized in Table 2. Additionally, the decrease rate due to the radionuclide migration (i.e., *AF* and *LF*) was calculated as the difference between the decrease rates of Simulation_Decay and the other models (Table 2). The decrease rate due to radiocesium migration in Dataset_Unpaved was −0.17 year^−1^. Although this value included both the *AF* and *LF*, it was smaller than the *AF* (−0.22 year^−1^) and *LF* (−0.20 year^−1^ as the difference between Simulation_AF and _Unpaved) of the simulations. It is difficult to assume the vertical penetration on paved surfaces. Thus, the decrease due to the radiocesium migration (−0.65 year^−1^) might represent the *LF*, and the value was also smaller than that in Europe (−0.81 year^−1^). These results suggested that both the penetration and wash-off of radionuclides in Fukushima were smaller than those in Europe.

**Table 2:** Decrease rates of the modeled and simulated norm-dH*(10) *dt*^−1^ in Fig. 3 during later phase from 60 days after the accident.

Results	Decrease rate (year^−1^)	Decrease rate due to radiocesium migrations (year^−1^)
Simulation_Decay	−0.26	
Simulation_AF	−0.48	−0.22
Simulation_Unpaved	−0.68	−0.42
Simulation_Paved	−1.07	−0.81
Dataset_Unpaved	−0.43	−0.17
Dataset_Paved	−0.91	−0.65

Decrease rate due to radiocesium migrations was calculated as the difference in the rate from that of Simulation_Decay.

Saito *et al*. reported that the decrease in the air dose rate due to the ground penetration of radiocesium in Fukushima was slower than that in Europe after the CNPP accident^16^, which is consistent with our results. Differences in soil properties could have been a reason for the slower penetration of radiocesium in Fukushima. The fixation of radiocesium by clay minerals is inhibited by organic matter in soil^28,29^, and it was observed that the relatively high proportion of organic matter in the soil increased the mobility of radiocesium in the Chernobyl area^30^. Faster penetration was locally observed in the coastal area in Fukushima than in the Chernobyl area, causing a discrepancy regarding the effect of organic matter^31^. However, the overall tendency of penetration in the scale of Fukushima’s terrain was possibly faster than that in the Chernobyl area because the latter is dominated by peat soil^32^ while the major soil type in the former is weathered granite, which has a large affinity to radiocesium^33,34^.

Differences among regional climatic and topographic properties could also be a factor affecting the wash-off of radionuclides. However, the greater precipitation and steep slopes of Fukushima compared with the Chernobyl area^31,35,36^ meant an increase in the wash-off in the former, causing the discrepancy in the decrease trend of the norm-dH*(10) *dt*^−1^ observed in this study. A possible reason for the discrepancy could have been differences in the field conditions. The monitoring points in this study were around residential areas, which are relatively flat and open spaces. Minor radiocesium wash-off from flat spaces was observed around the FDNPP^37^. Therefore, the *LF* in the residential areas dominated by flat fields was probably not affected by precipitation, while the greater precipitation and steep slopes of Fukushima could increase the wash-off in catchment scale^38^. In addition, other factors, such as snow cover, ground freezing during winter, vegetation, and human activities like decontamination and agricultural practices, can also affect the wash-off of radiocesium, and the complicated relationship among these factors might have been reflected in the difference in the *LF* between Fukushima and Europe. Unfortunately, these factors were difficult to compare quantitatively due to insufficient data. Although further studies are required to clarify the factors affecting the radiocesium wash-off associated with the *LF*., these results suggest that the radiocesium wash-off in the flat fields in Fukushima was less than that in Chernobyl area.

### Effect on the cumulative norm-H*(10)

**Figure 5:**
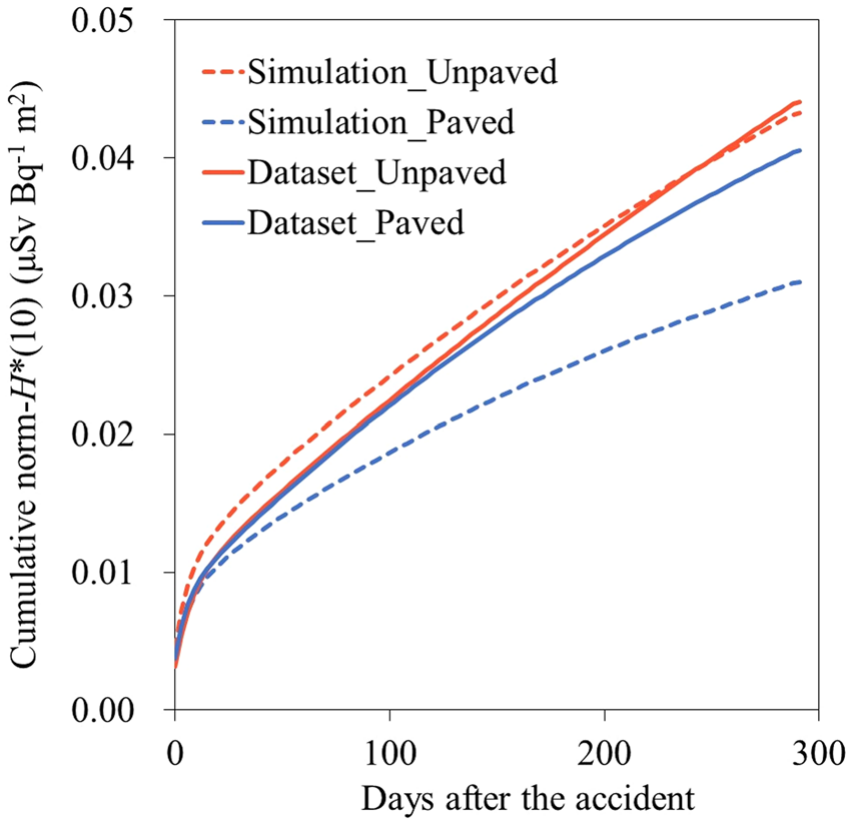
The cumulative norm-H*(10) calculated using models obtained in this study and simulations with parameters obtained in Europe after the CNPP accident.

To evaluate the effect of the difference in the decrease rates between the Fukushima and Chernobyl cases after the accident on the external dose, the modeled and simulated norm-dH*(10) *dt*^−1^ were cumulated (i.e., cumulative norm-H*(10)) through 2011 (Fig. 5). In the case of unpaved-dominant fields, the simulated and modeled values showed similar values at the end of 2011 because the overestimation of the cumulative norm-H*(10) during the initial period was offset by underestimation during the latter period due to the faster decrease in the norm-dH*(10) *dt*^−1^ in the simulation than in the model (Fig. 4). However, the cumulative norm-H*(10) simulated for paved-dominant fields was underestimated by approximately 24% of that modeled at the end of 2011 because of the faster decrease in the norm-dH*(10) *dt*^−1^ of the simulation during the later period. The *AF* and *LF* were regionally variable parameters, likely due to differences in the climatic and topographic properties, as shown in this study. The results suggested that their variations could have led to uncertainty regarding the external dose estimation.

## Methods

### Measurement

The monitoring of the dH*(10) *dt*^−1^ started on March 16, 2011, and the data collected until the end of 2011 were used in this study. Most data were measured using an NaI (Tl) scintillation survey meter (Hitachi Aloca Medical, Ltd., TCS-172B) calibrated according to the International Electrotechnical Commission’s standards (IEC 60846-1:2009). However, when high dH*(10) *dt*^−1^ values over 10 μSv h^−1^ were expected, some data were measured via an ionization-chamber-type survey meter (Hitachi Aloca Medical, Ltd., ICS-323C). The dH*(10) *dt*^−1^ was measured five times at each point, and the averaged value was logged.

### Identification of land types

To categorize the data into two datasets, the land types (e.g., paved and unpaved surfaces, buildings, etc.) within a radius of 10 m from the monitoring point were distinguished using ArcGIS 10.4 and the National Land Numerical Information service^39^. The land type details were also confirmed via satellite images provided by Google Earth.

### Data corrections

To evaluate the dH*(10) *dt*^−1^ derived from the FDNPP accident, the background was subtracted from the data before analysis. The Fukushima prefecture had a measured air kerma rate of 50 points from 1994 to 1999^40^. The average value was 0.042 μGy h^−1^ (standard deviation of 0.013 μGy h^−1^) and was applied as the background in this study.

Moreover, the dH*(10) *dt*^−1^ was normalized by the deposition amount of ^137^Cs on March 15, 2011. The deposition amount of ^137^Cs on July 01, 2011, was estimated from the results of the Third Airborne Monitoring Survey by MEXT^41^ as an average value in a circle with a 1 km diameter at the monitoring point. Then, the decay of ^137^Cs was corrected to match the value on March 15, 2011.

### Simulation of the norm-dH*(10) *dt*^−1^

Decreases in the norm-dH*(10) *dt*^−1^ were simulated due to decay, *AF*, and *LF* according to UNSCEAR^1^ with minor modifications as follows:

2 $${\rm{norm}}-d{H}^{\ast }(10)\,d{t}^{-1}(t)=\sum _{m}(\frac{{A}_{m}}{{A}_{Cs137}})\times exp(-{\lambda }_{m}\times t)\times {C}_{m}(0)$$

where *A*_m_ and *A*_Cs137_ are the initial deposition amounts of nuclide*s m* and ^137^Cs as the values on March 15, 2011. The *A*_m_ and *A*_Cs137_ values reported by UNSCEAR^1^ were applied in this study. Additionally, *λ*_m_ is the decay constant of the nuclides *m* (year^−1^), and *C*_m_(0) is the conversion coefficient (*C*_m_) from the deposition density of radionuclide *m* to the dH*(10) *dt*^−1^, assuming the initial relaxation mass depth of ^137^Cs. Saito *et al*.^16^ reported that the effective relaxation mass depth, which represents a practical exponential depth profile to reproduce the same air kerma rate at 1 m above the ground as that derived from the depth profile expressed by the hyperbolic secant function^42^, was 0.8 g cm^−2^ in June 2011. Based on the relationship between the *C*_m_ and relaxation mass depth reported by Saito and Petoussi-Henss^43^, this study estimated the *C*_m_(0) from the effective relaxation mass depth of 0.8 g cm^−2^. The *C*_m_(0) applied in this study is summarized in Table 3.

**Table 3:** Conversion coefficient from deposition density of radionuclide *m* to dH*(10) *dt*^−1^, when the relaxation mass depth is considered to be 0.8 g cm^−2^.

*m*	*C*_m_(0) (*μ*Sv h^−1^ per Bq m^−2^)
^131^I	1.59 × 10^−6^
^132^I	8.29 × 10^−6^
^132^Te	1.01 × 10^−6^
^134^Cs	5.84 × 10^−6^
^136^Cs	7.71 × 10^−6^
^137^Cs	2.15 × 10^−6^
^140^Ba	7.31 × 10^−7^
^110m^Ag	9.77 × 10^−6^
^129m^Te	1.42 × 10^−7^

Equation (2) represents the decrease in the norm-dH*(10) *dt*^−1^ due to decay only. Subsequently, the norm-dH*(10) *dt*^−1^ was multiplied by the *AF* and *LF* values of unpaved and paved surfaces according to UNSCEAR^1^ to represent the decreases due to the penetration and horizontal migration, respectively, of ^137^Cs. In the end, four simulation results were obtained in this study. The decreases in the norm-dH*(10) *dt*^−1^ were caused by: 1) decay only (Simulation_Decay); 2) decay and *AF* (Simulation_AF); 3) decay, *AF*, and *LF* on unpaved surfaces (Simulation_Unpaved); and 4) decay, *AF*, and *LF* on paved surfaces (Simulation_Paved).

## Supplementary information

Supplementary Information.

## Data Availability

The dataset we used in the study can be found in web site of Nuclear Regulation Authority^19^.
