# Supplementary material for: Initial decrease in the ambient dose equivalent rate after the Fukushima accident and its difference from Chernobyl
Source: Sci Rep. 2020 Mar 2;10:3859. doi: 10.1038/s41598-020-60847-0 (PMC7051962; doi:10.1038/s41598-020-60847-0)
Supplement: Supplementary file 1 — Supplementary Information. [file 41598_2020_60847_MOESM1_ESM.pdf]

# Initial decrease in ambient dose equivalent rate after the Fukushima accident and its difference from the Chernobyl.

Kazuya Yoshimura[1*], Jun Saegusa[2], Yukihisa Sanada[1]

[1]Fukushima Environmental Safety Center, Japan Atomic Energy Agency, 45−169 Sukakeba, Minamisoma, Fukushima 975−0036, Japan.

[2]Collaborative Laboratories for Advanced Decommissioning Science, Japan Atomic Energy Agency, 4−33, Muramatsu, Tokai−mura, Ibaraki 319−1194, Japan.

**Supplementary Table S1.** Descriptions of Dataset_Unpaved. The column of “No” indicates the monitoring sites numbered in the monitoring program by MEXT. The maximum and minimum $dH^{*}(10)\ dt^{-1}$ (μSv $hr^{-1}$) were observed during the monitoring period in 2011. Circle in the column of “Evacuation Zone” indicates the monitoring sites subjected to be evacuation zone at the end of 2011.

| No | Latitude | Longitude | Land type (proportion) | Monitoring period in 2011 | | | $dH^{*}(10)\ dt^{-1}$ (μSv $hr^{-1}$) | | $^{137}$Cs activity per unit area (kBq $m^{-2}$) | Evacuation zone |
|---|---|---|---|---|---|---|---|---|---|---|
| | | | | | | | Max. | Min. | | |
| 23 | 37°30'30.3" | 140°37'30.3" | Paved surface (50), Grassland (50) | March 16 | - | December 28 | 5.0 | 0.3 | 109 | |
| 31 | 37°33'56.2" | 140°44'37.8" | Paved surface (50), Grassland (20), Forest (30) | March 16 | - | December 28 | 66 | 4.7 | 1590 | ○ |
| 33 | 37°36'45.5" | 140°44'56.8" | Paved surface (50), Grassland (50) | March 16 | - | December 24 | 110 | 7.5 | 1938 | ○ |
| 37 | 37°45'18.5" | 140°41'17.1" | Paved surface (45), Grassland (15), Forest (40) | March 16 | - | December 24 | 4.7 | 2.2 | 436 | |
| 41 | 37°26'02.5" | 140°48'06.3" | Paved surface (50), Forest (50) | March 16 | - | December 28 | 13.0 | 0.4 | 169 | |
| 44 | 37°10'34.3" | 140°57'17.3" | Paved surface (40), Bare ground (20), Forest (40) | March 16 | - | December 28 | 19.0 | 0.2 | 94 | |
| 45 | 37°14'45.8" | 141°00'12.0" | Paved surface (40), Grassland (60) | March 16 | - | December 28 | 19.0 | 0.4 | 168 | ○ |
| 46 | 37°35'09.5" | 140°42'40.9" | Paved surface (50), Grassland (50) | March 17 | - | December 28 | 40 | 3.0 | 800 | ○ |
| 81 | 37°35'04.3" | 140°45'24.3" | Paved surface (50), Grassland (15), Forest (35) | March 24 | - | December 28 | 66 | 12 | 3002 | ○ |
| 83 | 37°33'37.5" | 140°49'26.0" | Grassland (100) | March 24 | - | December 28 | 110 | 28 | 4692 | ○ |
| 181 | 37°22'33.3" | 140°45'15.9" | Paved surface (35), Grassland (50), River (15) | May 20 | - | December 28 | 0.6 | 0.4 | 108 | |
| i10 | 37°44'15.3" | 140°42'13.1" | Paved surface (50), Grassland (50) | April 25 | - | December 24 | 5.5 | 3.3 | 690 | ○ |
| i20 | 37°40'26.4" | 140°46'41.4" | Paved surface (50), Grassland (50) | April 26 | - | December 22 | 7.0 | 4.7 | 821 | ○ |
| K3 | 37°31'23.9" | 140°44'52.6" | Paved surface (50), Grassland (50) | April 25 | - | December 22 | 1.9 | 1.3 | 318 | ○ |
| kw1 | 37°38'37.3" | 140°39'04.2" | Paved surface (50), Grassland (50) | April 26 | - | December 28 | 1.6 | 0.9 | 204 | |
| kw3 | 37°36'05.3" | 140°38'12.2" | Paved surface (50), Grassland (50) | April 26 | - | December 21 | 2.1 | 1.2 | 353 | ○ |
| n1 | 37°35'50.0" | 140°45'10.9" | Paved surface (40), Grassland (50), Forest (10) | April 27 | - | December 18 | 25 | 16 | 2709 | ○ |
| n2 | 37°34'37.4" | 140°46'45.6" | Paved surface (50), Grassland (50) | April 27 | - | December 18 | 24 | 15 | 3067 | ○ |
| n3 | 37°34'57.9" | 140°47'36.8" | Paved surface (50), Grassland (50) | April 27 | - | December 18 | 14 | 8.3 | 2544 | ○ |
| n8 | 37°33'18.5" | 140°46'44.9" | Paved surface (30), Forest (60), River (10) | April 27 | - | December 27 | 16 | 9.6 | 2306 | ○ |
| n10 | 37°33'37.4" | 140°42'29.2" | Paved surface (50), Grassland (50) | April 27 | - | December 27 | 2.2 | 1.3 | 387 | ○ |
| 111 | 37°17'51.7" | 140°45'33.3" | Paved surface (50), Forest (50) | May 20 | - | December 28 | 0.8 | 0.4 | 144 | |
| 113 | 37°27'34.1" | 140°45'09.4" | Paved surface (50), Grassland (50) | May 20 | - | December 28 | 1.5 | 0.8 | 205 | |
| 114 | 37°14'25.6" | 140°50'46.9" | Paved surface (40), Grassland (50), Forest (10) | May 23 | - | December 28 | 1.5 | 1.0 | 233 | |

11

**Supplementary Table S2.** Descriptions of Dataset_Paved.

| No. | Latitude | Longitude | Land type (proportion) | Monitoring period in 2011 | | | $dH^*(10)\,dt^{-1}$ ($\mu$Sv $hr^{-1}$) | | $^{137}$Cs activity per unit area (kBq $m^{-2}$) | Evacuation zone |
|---|---|---|---|---|---|---|---|---|---|---|
| | | | | | | | Max. | Min. | | |
| 1 | 37°45'02.1" | 140°28'00.7" | Building (100) | March 17 | - | December 24 | 8.5 | 0.3 | 186 | |
| 2 | 37°45'24.2" | 140°33'17.8" | Paved surface (80), Grassland (20) | March 17 | - | December 28 | 18 | 0.9 | 369 | |
| 4 | 37°39'45.0" | 140°35'44.2" | Paved surface (100) | March 17 | - | December 28 | 6.7 | 0.3 | 180 | |
| 5 | 37°47'26.9" | 140°55'48.0" | Paved surface (100) | March 17 | - | December 28 | 7.5 | 0.3 | 56 | |
| 7 | 37°42'00.2" | 140°57'45.4" | Paved surface (75), Grassland (25) | March 17 | - | December 28 | 5.0 | 0.3 | 82 | |
| 10 | 37°35'48.1" | 140°34'47.2" | Paved surface (75), Grassland (25) | March 17 | - | December 28 | 5.2 | 0.3 | 185 | |
| 22 | 37°30'52.4" | 140°39'17.6" | Paved surface (75), Grassland (25) | March 16 | - | December 28 | 26 | 0.2 | 129 | |
| 34 | 37°33'14.5" | 140°44'13.6" | Paved surface (75), Forest (25) | March 16 | - | December 28 | 25 | 3.4 | 738 | ○ |
| 38 | 37°07'27.9" | 140°56'52.8" | Paved surface (70), Grassland (30) | March 16 | - | December 28 | 2.5 | 0.2 | 93 | |
| 79 | 37°33'33.5" | 140°45'34.9" | Paved surface (90), Building (10) | March 20 | - | December 30 | 39 | 5.9 | 1838 | ○ |
| 102 | 37°44'07.7" | 140°36'34.0" | Paved surface (50), Building (10), Grassland(40) | April 7 | - | December 28 | 1.7 | 0.3 | 228 | |
| 104 | 37°30'11.6" | 140°45'49.8" | Paved surface (60), Forest (10), River (30) | April 7 | - | December 28 | 3.4 | 0.6 | 381 | ○ |
| 112 | 37°13'10.1" | 140°57'04.7" | Paved surface (65), Gravel (20), Forest (15) | May 21 | - | December 28 | 1.2 | 0.5 | 129 | |
| i9 | 37°43'00.3" | 140°41'28.3" | Paved surface (60), Building (15), Grassland (25) | April 25 | - | December 24 | 5.9 | 3.4 | 783 | ○ |
| K11 | 37°28'51.8" | 140°48'15.4" | Paved surface (60), Forest (40) | April 25 | - | December 24 | 2.1 | 1.3 | 289 | ○ |
| ni2 | 37°34'47.1" | 140°40'30.6" | Paved surface (50), Building (25), Gravel (25) | May 4 | - | December 28 | 1.0 | 0.6 | 208 | |
